# The expression of mercaptopyruvate sulfurtransferase serves as a potential biomarker for prognostic stratification and immunotherapy in certain cancers

**DOI:** 10.3389/fonc.2025.1686443

**Published:** 2026-01-14

**Authors:** Boran Cui, Chenchen Xia, Li Yang, Xiaoyu Xi, Xinxin Gong, Yixi Liu, Cai Tian, Xiaoli Du, Jiexian Du, Zengfang Hao

**Affiliations:** 1 Department of Gynecology, The Second Hospital of Hebei Medical University, Shijiazhuang, Hebei, China; 2 Hebei Medical University, Shijiazhuang, Hebei, China; 3 Department of Gynecology, Xingtai Women and Children's Health, Xingtai, Hebei, China; 4 Department of Gynecology, Peking University People's Hospital, Beijing, China; 5 Hebei North University, Zhangjiakou, Hebei, China; 6 Shandong First Medical University, Jinan, Shandong, China; 7 Department of Gynecological Ultrasound, The First Hospital of Hebei Medical University, Shijiazhuang, Hebei, China; 8 Department of Gynecology, Shijiazhuang Hospital of Traditional Chinese Medicine, Shijiazhuang, Hebei, China; 9 Department of Pathology, The Second Hospital of Hebei Medical University, Shijiazhuang, Hebei, China

**Keywords:** immune infiltration, immunohistochemistry, methylation, MPST, pan-cancer, prognosis

## Abstract

**Introduction:**

3 - Mercaptopyruvate Sulfurtransferase (3 - MST) is an enzyme encoded by the Mercaptopyruvate Sulfurtransferase (MPST) gene. The enzyme is associated with a variety of cancers such as lung adenocarcinoma. However, there is a lack of comprehensive pan-cancer analysis on the potential impact of MPST on cancer diagnosis, prognosis, and immune response.

**Methods:**

Based on data from The Cancer Genome Atlas and the Human Protein Atlas, we conducted a comprehensive bioinformatic analysis to investigate the oncogenic role of MPST. Immune-related characteristics and tumor microenvironment infiltration were assessed using TIMER2.0. Protein-protein interaction networks were constructed via STRING, while DNA methylation differences across cancers were analyzed with UALCAN. Further insights into genetic alterations and functional states at the single-cell level were obtained from cBioPortal and CancerSEA. Finally, clinical samples were collected and subjected to immunohistochemical staining to preliminarily validate the bioinformatic findings.

**Result:**

The research assessed the links between MPST levels and various cancer outcomes, genetic alterations, immune cell presence, and DNA methylation patterns. Single-cell sequencing was utilized to deepen our comprehension of its functional importance. Furthermore, the unique immunohistochemical profiling of MPST in patient tissue was confirmed. Significant expression differences between tumors and normal tissues in certain cancer types suggest a correlation between MPST expression and clinical outcomes. Furthermore, correlations exist between MPST expression levels in different immune cells and the tumor immune microenvironment. Our observations also indicate a role for MPST methylation, along with the advantageous effects of highly amplified mutations and deletions of the MPST gene.

**Discussion:**

Significantly, MPST shows potential for early cancer identification and as a predictive factor for various tumor forms.

## 1 Introduction

Cancer persists as a perennial global health challenge, characterized through unrelenting escalation in both incidence and mortality burdens (1). While therapeutic strategies have evolved from radiotherapy and chemotherapy to targeted approaches and immunotherapy, progressively deepening our understanding of tumor pathogenesis and enhancing clinical outcomes. However, the efficacy of immunotherapy necessitates further validation across diverse malignancies. Pan-cancer analysis involves the systematic examination of gene expression patterns across multiple malignancies, enabling comparative assessment of molecular similarities and differences among tumor types.

Within the mammalian body, cystathionine β -synthetase (CBS), cystathionine γ -lyase (CSE), and mercaptopyruvate sulfurtransferase (MPST) are key enzymatic regulators of endogenous hydrogen sulfide (H_2_S) biosynthesis. Together, these enzymes coordinate the production of H_2_S in the mammalian system. As a gas signaling molecule, H_2_S works together with nitric oxide and carbon monoxide and is widely distributed in various physiological systems of mammals (2, 3). Although CSE, CBS, and MPST are members of the same enzyme superfamily, they demonstrate considerable variation in their structural and functional properties. These differences encompass active site architecture, cofactor and substrate specificity, catalytic mechanisms, regulatory controls, and subcellular localization, which collectively influence their distinct roles in physiological processes within the cardiovascular and nervous systems (4, 5). Among them, the roles of CSE and CBS have been extensively studied. However, the specific functions and regulatory mechanisms of MPST, particularly in the context of tumor biology, remain less clearly defined and warrant further investigation.

Mercaptopyruvate sulfurtransferase (MPST) is a key enzyme responsible for endogenous hydrogen sulfide (H_2_S) production, as evidenced by studies demonstrating its essential role in H_2_S-mediated physiology (6). Beyond this central biochemical function, its expression pattern has emerged as a potential prognostic marker in various cancers. The activation of MPST is involved in tRNA sulfation, protein aminoacylation, and detoxification of cyanide (7). It uses substrate 3-mercaptopyruvate to transamine l-cysteine, glutamic acid oxaloacetate transaminase, or d-cysteine metabolism mediated by d-amino acid oxidase (7, 8). A study on the expression of MPST in colorectal cancer showed that decreased MPST expression in colorectal cancer patients was associated with disease progression, making this enzyme a potential tumor marker for colon malignancies (9). Research on hepatocellular carcinoma has also shown that MPST can serve as a tumor suppressor and a biomarker to predict patient prognosis (10). In glioblastoma cells, high production of reactive oxygen species (ROS) and MPST activity combine to promote cancer cell movement (11). Cancer cells exhibiting elevated levels of MPST have been observed to demonstrate enhanced cytoprotective capabilities and a greater propensity for metastasis. Furthermore, genetic deletion of MPST in bacterial, zebrafish, and mammalian models results in heightened oxidative stress and increased susceptibility to oxidants, indicating that the antioxidant function of MPST is evolutionarily conserved (12, 13). Loss-of-function mutations in the MPST gene result in mercaptolactate-cysteine disulphuria, a hereditary metabolic disorder marked by neurodevelopmental deficits in humans. Consistent with this pathogenic mechanism, murine MPST knockout models display validated anxiety-like behavioral phenotypes (14). Moreover, we found that it is overexpressed in many different types of cancer, suggesting that it has a certain role in differential diagnosis. In this study, we further developed the value of MPST in immunotherapy and diagnosis through the Bioinformatics tool, which opened a new idea for future immunotherapy.

## 2 Materials and methods

### 2.1 Data collection and processing

Transcriptomic profiles and clinical metadata encompassing 33 tumor types were accessed via The Cancer Genome Atlas (TCGA; https://portal.gdc.cancer.gov). Corresponding normal/neoplastic tissue immunohistochemical data originated from Human Protein Atlas (HPA; https://www.proteinatlas.org) (15, 16). Subsequent GEPIA2 analysis (http://gepia2.cancer-pku.cn) pinpointed the 100 strongest MPST-correlated genes across TCGA datasets.

### 2.2 Collection of clinical samples

This study procured matched specimens from The Second Hospital of Hebei Medical University, including: nine bladder urothelial carcinomas (BLCA), nine rectal adenocarcinomas (READ), nine testicular germ cell tumors (TGCT), and corresponding adjacent normal tissues. All specimens were preserved using a tissue-specimen fixative, followed by cell isolation and culture procedures. None of the patients had undergone any treatment prior to surgery. Ethics committee approval enabled documented consent acquisition from all participants.

### 2.3 Analysis of MPST differential expression, prognosis, and evaluation of nomogram models

MPST mRNA expression differences in tumor tissues and paired normal tissues underwent stratified analysis using paired and unpaired approaches. Supplementary immunohistochemical analysis of MPST protein distribution was performed using the HPA database. The relationship between MPST levels and clinical outcomes [overall survival (OS), progression-free interval (PFI), and disease-specific survival (DSS)] was assessed by a Log-rank test in the TCGA dataset. Receiver operating characteristic (ROC) curves were also generated for tumor types that showed MPST correlation (16). To determine whether the risk scoring system is an independent prognostic factor, univariate and multivariate Cox proportional hazards regression analyses were performed on relevant clinical pathology variables. The “rms” package integrated independent prognostic factors into nomograms predicting 1-, 3-, and 5-year overall survival (OS) probabilities. Nomogram discrimination employed ROC analysis with calibration plots (17). We further examined links between MPST expression and key clinical variables including gender, N stage, and pathological stage (16).

### 2.4 Analysis of the immune infiltration

To investigate MPST-tumor immune microenvironment relationships, we employed TIMER2.0 (http://timer.cistrome.org) analyzing immune infiltration-MPST expression correlations across TCGA malignancies. Focus included B cells, macrophages, CD4+ and CD8+ T cells (16). Additionally, the ESTIMATE R package (version 1.0.13) was employed to calculate stromal, immune, and ESTIMATE scores for each tumor sample based on gene expression data, enabling quantification of the tumor microenvironment composition. Furthermore, publicly available data on tumor mutation burden (TMB) and microsatellite instability (MSI) were downloaded and analyzed to evaluate their correlations with MPST expression in a pan-cancer context (18–20). To further investigate the immunomodulatory role of MPST, we conducted a comprehensive correlation analysis between MPST expression and a panel of 60 immune checkpoint genes, categorized into 24 inhibitory and 36 stimulatory genes, using Pearson correlation across multiple cancer types (19, 21, 22). Patient stratification by MPST levels evaluated immune infiltration effects on overall survival across cancer types.

### 2.5 Protein-protein interaction network analysis and functional enrichment analysis

After analyzing the differential expression of MPST in different tumor types, we performed gene set enrichment analysis (GSEA) via clusterProfiler. The top 100 genes most related to MPST expression patterns were obtained from the Interactive Gene Expression Profile Analysis 2 (GEPIA2) database. Protein interaction (PPI) networks were constructed and visualized using Cytoscape (v3.7.2) and the STRING database. The network was constructed using 100 genes related to MPST to identify significant interactions (23). Functional characterization leveraged gene ontology (biological processes, cellular components, molecular functions) and Kyoto Encyclopedia of Genes and Genomes enrichment analyses (24, 25).

### 2.6 Single-cell sequencing

CancerSEA is a database dedicated to single cell sequencing, which provides in-depth understanding of various functional states of cancer cells at single cell resolution. Single-cell sequencing enables essential functional characterization of target biomolecules. Leveraging this pivotal technology, we explored MPST expression links to diverse tumor functionalities. A t-distributed random neighborhood embedding (t-SNE) plot was also generated to depict the expression profile of MPST in individual cells from TCGA samples (26, 27).

### 2.7 Methylation

UALCAN (http://ualcan.path.uab.edu) is an interactive network platform for comprehensive analysis of TCGA gene expression data. In this study, we used UALCAN to detect DNA methylation levels in cancers (28). Additionally, we also used cBioPortal (http://www.cbioportal.org), a platform that contains TCGA multi-dimensional tumor genomic data, to analyze data from 32 cancer types for more in-depth research.

### 2.8 Analysis of the genetic alteration

We retrieved alteration frequency, mutation types, mutation sites, and 3D structural data for candidate proteins across all TCGA tumor samples utilizing the cBioPortal database (https://www.cbioportal.org/). This data served to evaluate the prognostic impact of genomic alterations affecting MPST genes, with *P* < 0.05 indicating statistical significance.

### 2.9 Immunohistochemistry

Perform IHC staining according to the previously described protocol. In short, dewaxing and dehydration of glass slides were performed, followed by thermally induced antigen repair of MPST using PBS (pH 7.4). We first used 3% hydrogen peroxide solution to eliminate the capability of endogenous peroxidase, then added 3% bovine serum albumin (BSA) drop by drop to tissue sections to achieve uniform serum blocking. Next, the sections were incubated with primary antibodies targeting MPST at night. Following PBS washes, tissues underwent 30-minute room temperature incubation with horseradish peroxidase-conjugated secondary antibodies matched to the primary antibody. Following additional PBS washes, freshly prepared diaminobenzidine solution was added drop by drop to the tissue for color development and the reaction was terminated by rinsing with tap water (23). Following hematoxylin counterstaining, tissue sections underwent dehydration in a graded ethanol series, clearing in xylene, and mounting prior to microscopic examination.

### 2.10 Statistical analysis

We performed statistical analyses and bioinformatics utilizing R software (v4.2.1) and public bioinformatics databases. Group comparisons used either Wilcoxon rank-sum or t-tests based on data characteristics. Survival outcomes were assessed via Kaplan-Meier analysis with log-rank testing, while Cox proportional hazards regression derived hazard ratios and 95% confidence intervals (CIs). We initially conducted univariate regression analyses to evaluate the association between common clinical factors and the outcome. Variables demonstrating a statistical trend towards significance (*P* < 0.1) in these univariate analyses, and deemed theoretically relevant, were selected for inclusion in the subsequent multivariable models. The final prognostic model incorporated only those variables that retained statistically significant associations within the multivariable analysis. The final prognostic model retained only those variables demonstrating statistically significant associations in the multivariable context. P-values were adjusted for multiple hypothesis testing using the False Discovery Rate (FDR) method, and findings with an FDR < 0.05 were considered statistically significant.

## 3 Result

### 3.1 MPST expression in the pan-cancer

**Figure 1:**
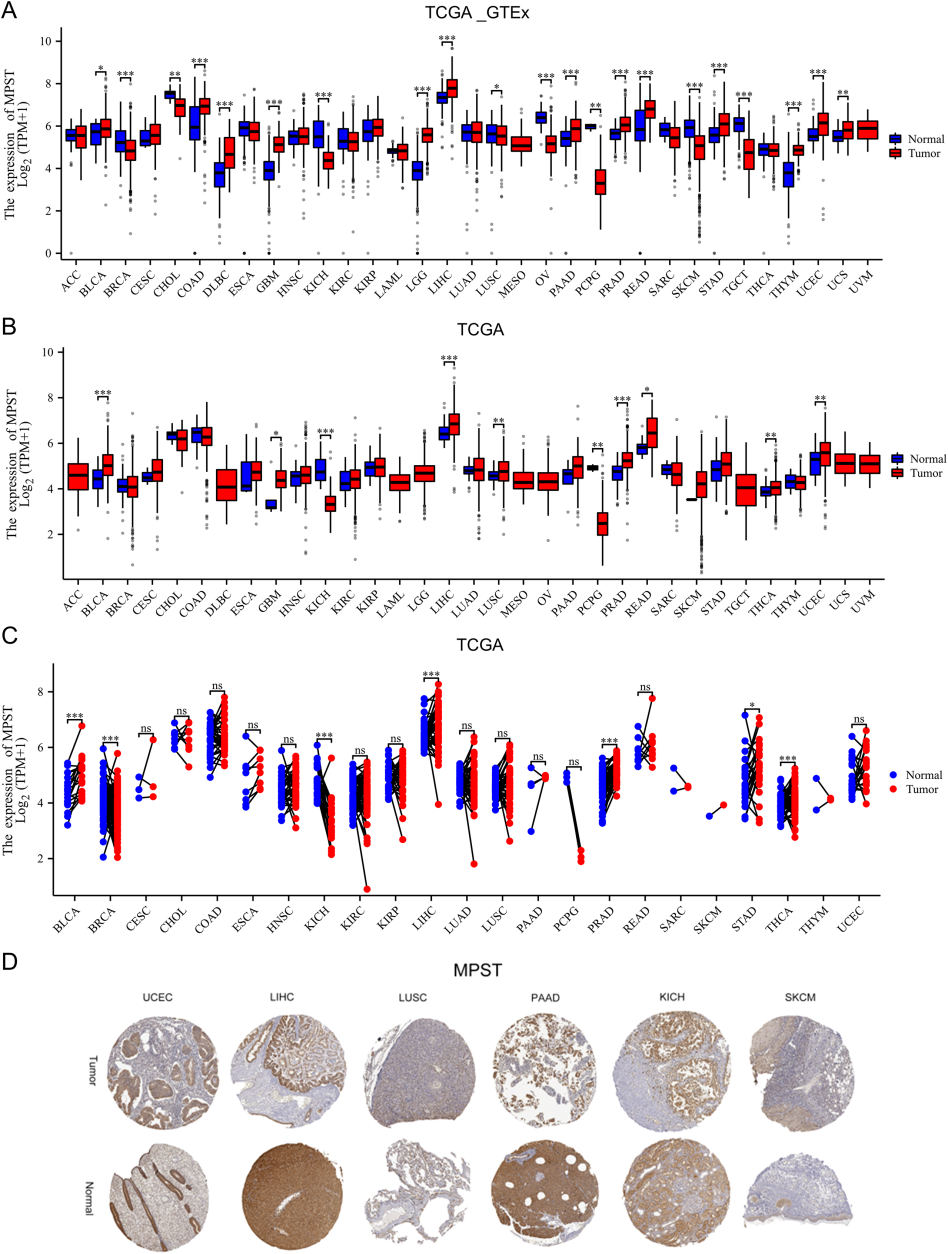
Expression of MPST in the pan-cancer **(A)** Expression of MPST mRNA in the 33 tumors in the TCGA _ GTEx samples. **(B)** MPST mRNA expression in 33 tumors from the TCGA database. **(C)** Expression of MPST in the 18 tumor-paired samples from the TCGA database. (ns, *P* > 0.05; *P < 0.05; **P < 0.01, ***P < 0.001). **(D)** Immunohistochemical images of MPST in normal tissues and tumor tissues taken from HPA.

Leveraging on TCGA data, MPST expression levels were compared across 33 cancer types (**Figure 1A**). The findings indicated that MPST expression varied across the majority of tumor types, demonstrating both elevated and reduced levels (**Figure 1B**). MPST exhibited marked upregulation in 8 of the 33 tumor types, specifically bladder urothelial carcinoma (BLCA), glioblastoma multiforme (GBM), liver hepatocellular carcinoma (LIHC), lung squamous cell carcinoma (LUSC), prostate adenocarcinoma (PRAD), rectum adenocarcinoma (READ), uterine corpus endometrioid carcinoma (UCEC), and thyroid carcinoma (THCA). Additionally, we analyzed 23 common paired tumor samples for MPST expression (**Figure 1C**). MPST exhibited significant elevation in 8 among 33 tumor types, specifically bladder urothelial carcinoma (BLCA), liver hepatocellular carcinoma prostate (LIHC), adenocarcinoma (PRAD), stomach adenocarcinoma (STAD), thyroid carcinoma (THCA). Furthermore, utilizing the Human Protein Atlas (HPA), MPST expression was evaluated in non-neoplastic and cancerous tissues across multiple human organs, and extracted relative immunohistochemical images of normal tissues from the uterus, liver, lung, pancreas, skin, kidney and tumor tissues (**Figure 1D**). Since MPST has been demonstrated to be associated with immunoinvasive and prognostic effects in endometrial cancer, our subsequent study focused exclusively on other cancers with significant differential expression (23).

### 3.2 Association between MPST expression and prognosis across multiple cancer types

**Figure 2:**
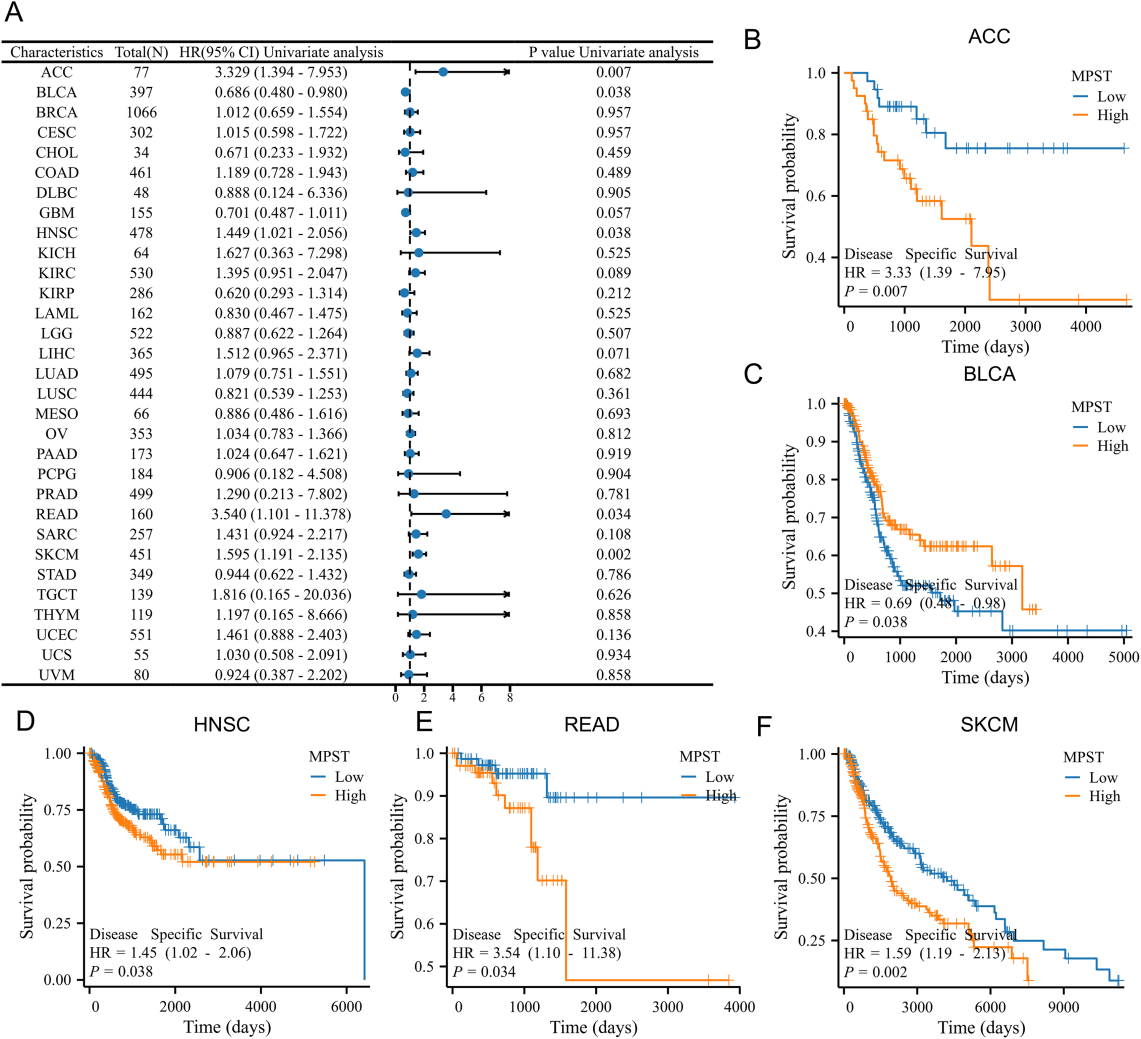
The relation between MPST expression and DSS in pan-cancer. **(A)** Effect of MPST expression on the pan-cancer DSS. **(B–F)** Effect of MPST expression on DSS in ACC, BLCA, HNSC, READ, and SKCM, respectively.

To get a handle on whether MPST expression could be a useful indicator of prognosis across various cancers, we utilize Kaplan-Meier survival analyses to see how it lined up with clinical prognosis. We first analyzed MPST expression’s association with overall survival (OS) (**Supplementary Figure S1A**). Elevated MPST levels correlated with poorer OS in ACC, SARC, and SKCM (**Supplementary Figures S1B–F**). Next, we investigated MPST’s link to disease-specific survival (DSS) (**Figure 2A**). High expression predicted worse DSS in ACC, HNSC, READ, and SKCM, but better DSS in BLCA (**Figures 2B–F**). Finally, assessment of progression-free interval (PFI) (**Supplementary Figure S2A**) showed increased MPST associated with negative PFI in ACC, SKCM, and TGCT, and postive PFI in BLCA and GBM (**Supplementary Figures S2B–F**). Furthermore, ROC curve analyses were conducted for six tumor types exhibiting prognostic relevance to MPST expression (**Supplementary Figures S3A–F**), indicating that MPST possesses high diagnostic accuracy in BLCA, GBM, HNSC, READ, SARC, and SKCM.

### 3.3 Correlation between MPST expression and clinical parameters

**Figure 3:**
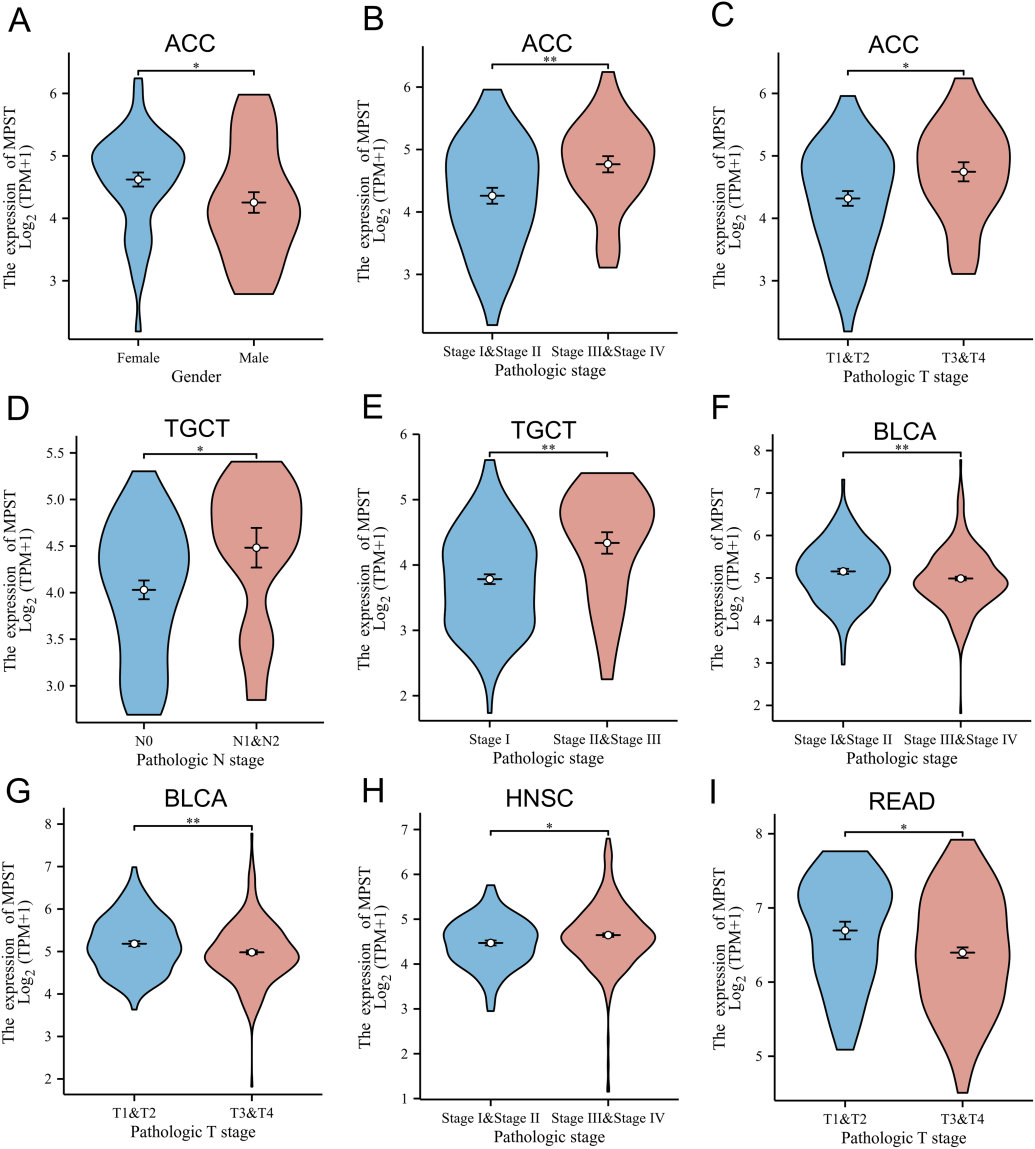
Correlation between MPST expression and clinicopathological parameters. **(A)** In ACC, MPST expression co-associated with sex. **(B-D)** MPST expression in ACC, BLCA and READ. **(E)** Expression of MPST is correlated with the N phase of TGCT. **(F–I)** MPST expression is correlated with the pathological stages of ACC, BLCA, HNSC, and TGCT. (*P < 0.05; **P < 0.01).

Analysis across 33 TCGA tumor types revealed six cancers (UCEC, BLCA, SKCM, SARC, ACC, HNSC) with MPST expression linked to prognosis. We then investigated MPST’s association with clinicopathological features in these cancers. Results showed MPST expression correlated with: sex in ACC (**Figure 3A**); tumor size in BLCA and READ (**Figures 3G, I**); lymphatic metastasis in TGCT (**Figure 3D**); and pathological stage in ACC, BLCA, HNSC, and TGCT (**Figures 3B, E, F, H**). In READ, survival analysis revealed a significant association: lower MPST expression was correlated with longer disease-specific survival (**Figure 2E**). However, this favorable survival outcome contrasted with the concurrent observation that lower MPST expression was also associated with more advanced T-stage (**Figure 3I**), a feature typically linked to worse prognosis. This presents an unresolved clinical association, indicating that the role of MPST in READ is complex and may not be directly inferred from conventional clinicopathological parameters.

### 3.4 Nomogram model development and validation

Nomogram development leveraged univariate Cox regression outcomes per tumor type (**Tables 1**, **2**), generating tumor-specific predictive models. The prognostic significance of these models was subsequently validated, and their predictive accuracy for overall survival (OS) at 1, 3, and 5 years was assessed through calibration curves. Findings indicated that within the nomogram model, MPST was substantially contributed to the prognosis and demonstrated robust predictive performance for ACC and SARC OS (**Supplementary Figures S4A, C**), Furthermore, the calibration plots for 1-, 3-, and 5-year survival predictions confirmed the high accuracy of the nomogram models in forecasting OS (**Supplementary Figures S4B, D**).

**Table 1:** Univariate and multivariate Cox regression analysis between ACC.

Characteristics	Total (N)	Univariate analysis		Multivariate analysis	
		Hazard ratio (95% CI)	*P* value	Hazard ratio (95% CI)	*P* value
Age	79				
> 50	38	Reference			
<= 50	41	0.556 (0.262 - 1.182)	0.127		
Pathologic stage	77				
Stage I&Stage II	46	Reference		Reference	
Stage III&Stage IV	31	6.476 (2.706 - 15.498)	**< 0.001**	2.290 (0.638 - 8.219)	0.204
Residual tumor	70				
R0	55	Reference		Reference	
R1&R2	15	12.617 (5.064 - 31.434)	**< 0.001**	7.149 (2.103 - 24.299)	**0.002**
MPST	79				
Low	39	Reference		Reference	
High	40	2.725 (1.228 - 6.045)	**0.014**	2.594 (1.024 - 6.567)	**0.044**

Bold values indicate statistical significance at P<0.05.

**Table 2:** Univariate and multivariate Cox regression analysis between SARC.

Characteristics	Total (N)	Univariate analysis		Multivariate analysis	
		Hazard ratio (95% CI)	*P* value	Hazard ratio (95% CI)	*P* value
Age	263				
<= 60	130	Reference			
> 60	133	1.285 (0.864 - 1.911)	0.216		
Gender	263				
Female	144	Reference			
Male	119	0.905 (0.607 - 1.349)	0.623		
Tumor multifocal	239				
No	199	Reference		Reference	
Yes	40	2.402 (1.502 - 3.840)	**< 0.001**	2.262 (1.307 - 3.914)	**0.004**
Tumor depth	209				
Superficial	21	Reference		Reference	
Deep	188	2.888 (0.910 - 9.168)	0.072	2.570 (0.800 - 8.250)	0.113
MPST	263				
Low	131	Reference		Reference	
High	132	1.527 (1.022 - 2.282)	**0.039**	1.696 (1.047 - 2.748)	**0.032**

Bold values indicate statistical significance at P<0.05.

### 3.5 Correlation of MPST expression with the tumor immune microenvironment

**Figure 4:**
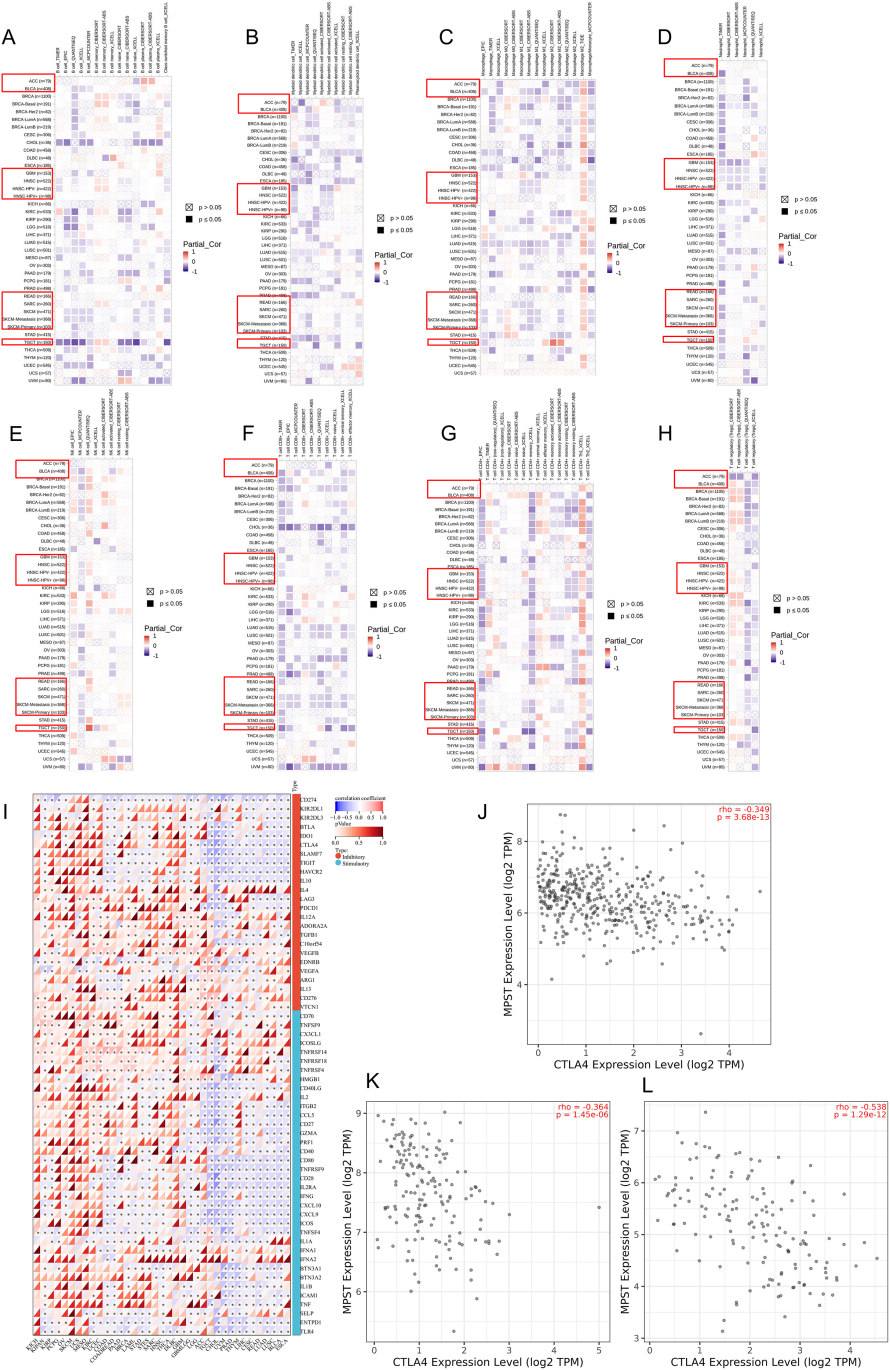
Correlation between MPST expression and tumor immune microenvironment. **(A)** heat map between MPST expression and B cells, **(B)** DC cells, **(C)** macrophages, **(D)** neutrophils, **(E)** NK cells, **(F)** T cells CD4+, **(G)** T cells CD8+, **(H)** and regulatory T cells. **(I)** Heatmap summarizing the Pearson correlation coefficients between MPST expression and the mRNA levels of key immune checkpoint genes in various cancer types from TCGA. Scatter plots validating the correlation between MPST and CTLA-4 expression in three representative cancer types: **(J)** BLCA, **(K)** READ, and **(L)** TGCT. Tumor types with a previously established prognostic association with MPST are highlighted with a red box.

**Figure 5:**
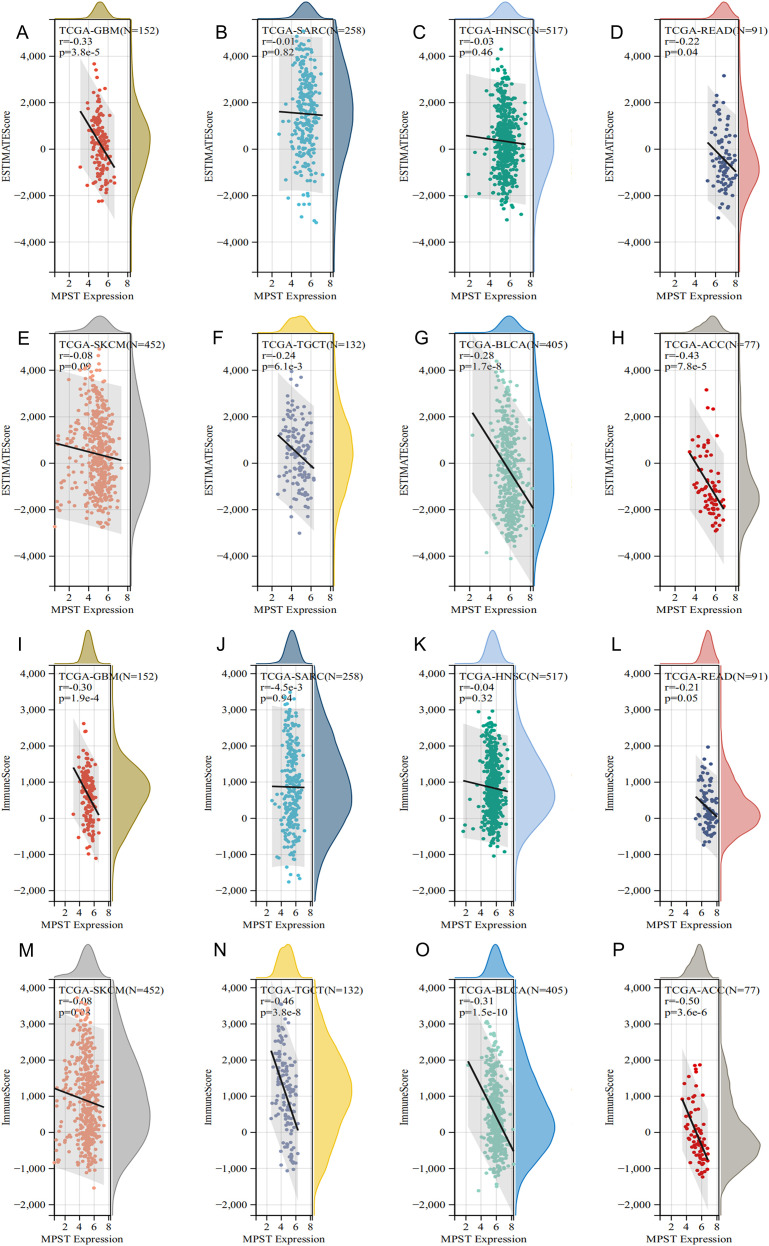
Associations between MPST expression and immune infiltration across multiple cancer types (GBM, SARC, HNSC, READ, SKCM, TGCT, BLCA, ACC) as determined by the ESTIMATE algorithm. **(A–H)** Correlations with the ESTIMATE score; **(I–P)** Correlations with the Immune score.

As the tumor immune microenvironment critically influences tumorigenesis, we investigated pan-cancer associations between MPST expression and immune characteristics using TIMER2.0. This database analyzed correlations connects MPST levels with immune cell infiltration, with heatmaps revealing relationships for: B cells, dendritic cells, macrophages, neutrophils, NK cells, CD8+ T cells, CD4+ T cells, and regulatory T cells (**Figures 4A–H**). Furthermore, we employed the ESTIMATE algorithm to calculate immune and stromal scores. This algorithmic analysis revealed that MPST expression was negatively correlated with the ESTIMATE, Immune, and Stromal scores in five of the eight prognosis-related cancer types: GBM, READ, TGCT, BLCA, and ACC (**Figure 5**, **Supplementary Figures S5A–H**).

### 3.6 Algorithmic assessment of the tumor immune microenvironment: tumor mutational burden, microsatellite instability, and immune checkpoints

To further computationally investigate the potential role of MPST in the tumor immune microenvironment, we analyzed its predicted association with tumor mutational burden (TMB) and microsatellite instability (MSI). Our algorithmic analysis indicated a positive correlation between MPST expression and TMB in most studied cancers, except SKCM (**Supplementary Figure S5I**). Regarding MSI, MPST expression showed an association with MSI status across cancers (**Supplementary Figure S5J**). Additionally, a pan-cancer correlation analysis suggested that MPST expression may be linked to the transcript levels of several immune checkpoint molecules, including PD-L1 and CTLA-4 (**Figure 4I**). Focusing on CTLA-4 as a representative, we validated significant negative correlations in the predictions for BLCA, TGCT, and READ (**Figures 4J–L**). These in silico findings highlight that the predicted relationships between MPST and immune features vary by cancer type.

### 3.7 Overall survival effect of MPST expression connected with immune infiltration

**Figure 6:**
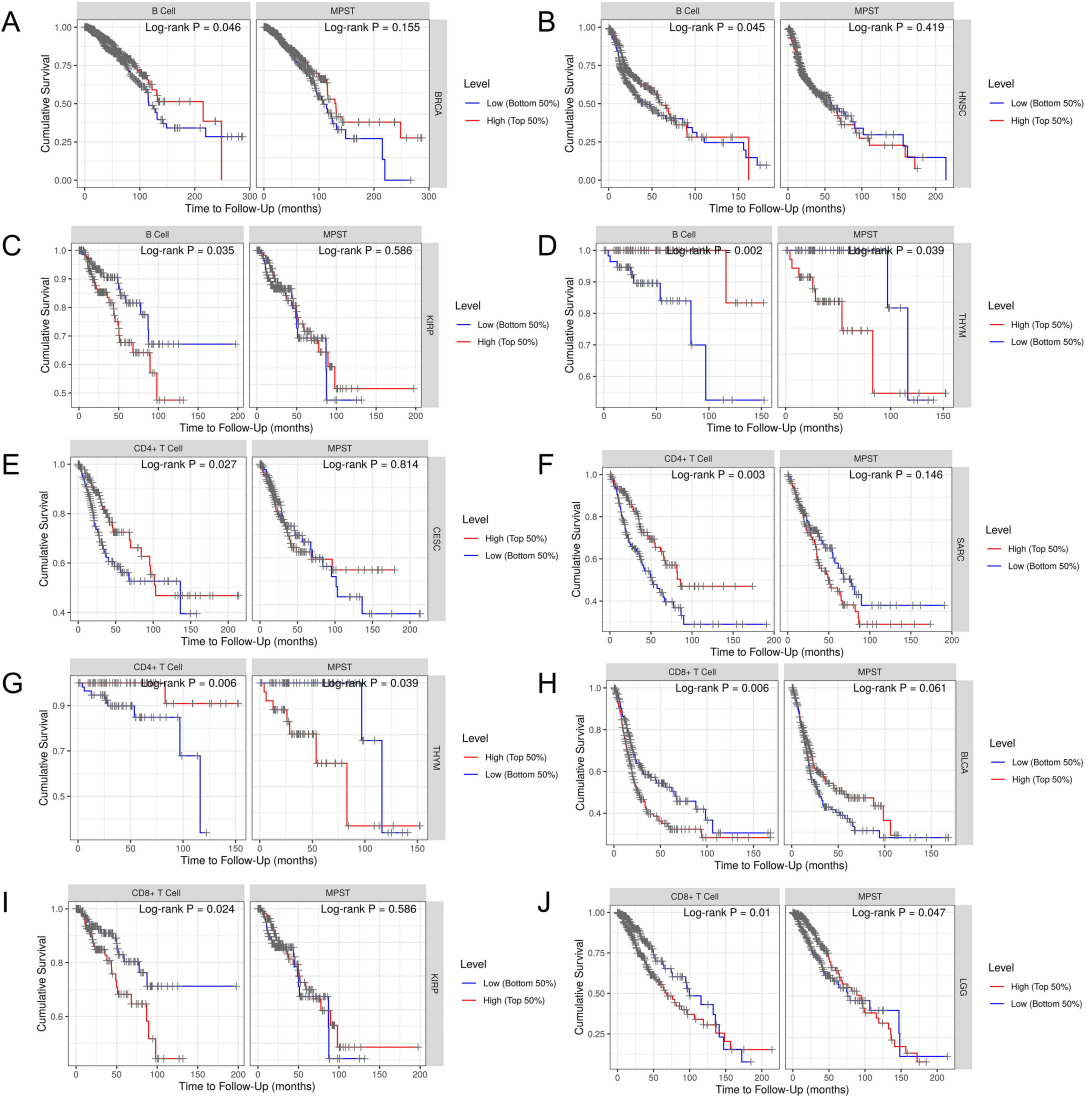
Combined with the expression of MPST and immune cell infiltration, B cell infiltration affected the prognosis of **(A)** BRCA, **(B)** HNSC, **(C)** KIRP and **(D)** THYM. CD4+ T cell infiltration was associated with OS in **(E)** CESC, **(F)** SARC, and **(G)** THYM. CD8+ T cell infiltration was associated with OS in **(H)** BLCA, **(I)** KIRP and **(J)** LGG.

We quantified MPST-associated immune infiltration effects on patient survival trajectories. Analysis of the combined impact of MPST expression and immune cell infiltration revealed significant effects on prognosis in BRCA (**Figure 6A**), HNSC (**Figure 6B**), KIRP (**Figure 6C**), and THYM (**Figure 6D**). Specifically, concurrent MPST expression and elevated B cell infiltration correlated with improved survival in BRCA. Furthermore, CD4+ T cell infiltration demonstrated an association with overall survival (OS) in CESC (**Figure 6E**), SARC (**Figure 6F**), and THYM (**Figure 6G**), while CD8+ T cell infiltration was linked to OS outcomes in BLCA (**Figure 6H**), KIRP (**Figure 6I**), and LGG (**Figure 6J**). Prognostically, patients exhibiting high B cell proportions showed better outcomes in BRCA, HNSC, and THYM; similarly, high CD4+ T cell proportions were favorable in CESC, SARC, and THYM. Conversely, low CD8+ T cell proportions was correlated with increased prognosis in BLCA, KIRP, and LGG.

### 3.8 Protein-protein interaction network and functional enrichment analysis of MPST related genes

**Figure 7:**
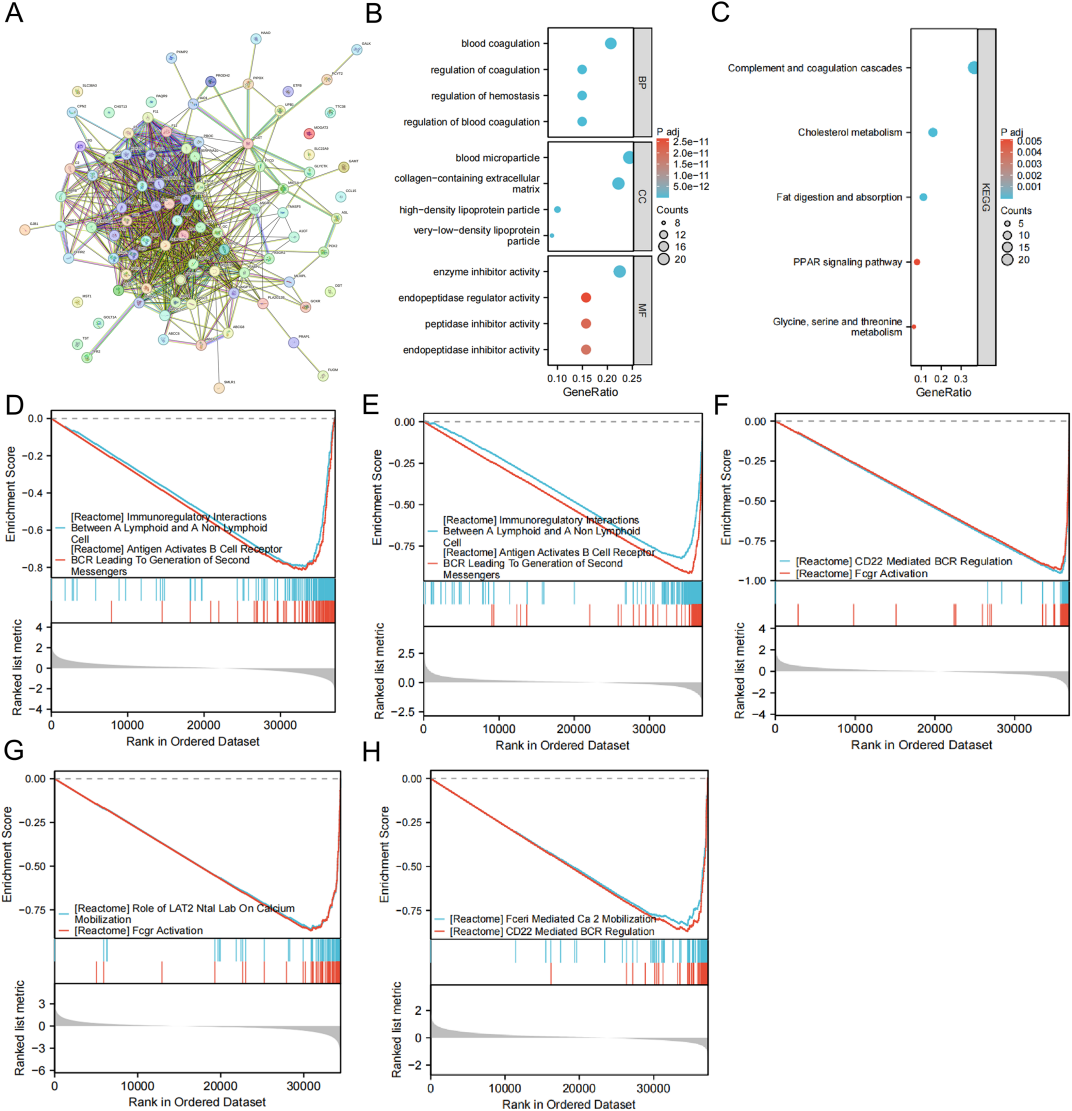
Functional enrichment analysis of the genes associated with MPST. **(A)** A PPI network based on the 100 MPST-related genes. **(B)** GO enrichment analysis based on the 100 MPST-related genes (including BP, CC, and MF). **(C)** KEGG pathway analysis based on the 100 MPST-related genes. **(D–H)** GSEA is based on the differential expression analysis of BLCA, SKCM, SARC, ACC and HNSC, respectively.

Following identification of the 100 most MPST-linked genes, PPI networks revealed their functional interplay, which is generated by STRING database (**Figure 7A**).

For MPST, the GO analysis (**Figure 7B**) showed the top several biological processes including “coagulation regulation”, “hemostasis regulation”, “coagulation regulation” and “coagulation regulation”. The most enriched cellular components were “very low-density lipoprotein particles”, “blood particles”, “high density lipoprotein particles” and “collagen-containing extracellular matrix”. The most abundant molecular functions are “endopeptidase inhibitor activity”, “enzyme inhibitor activity”, “endopeptidase regulatory activity” and “peptidase inhibitor activity”. The results of KEGG pathway enrichment analysis indicate (**Figure 7B**), they are related to “glycine, serine and threonine metabolism”, “PPAR signaling pathway”, “fat digestion and absorption”, “cholesterol metabolism” and “complement and coagulation cascade”.

### 3.9 Enrichment analysis of the gene sets

We employed GSEA to characterize MPST’s biological functions in six tumors with prognostic relevance to MPST, informed by its expression divergence analysis. Those associated with MPST include BLCA (**Figure 7D**), SKCM (**Figure 7E**), SARC (**Figure 7F**), ACC (**Figure 7G**), HNSC (**Figure 7H**). The results indicate that MPST is mainly associated with translation elongation, immune-related pathways, Fcgr activation, as well as calcium mobilization in eukaryotes.

### 3.10 MPST expression pattern at the single-cell level

**Figure 8:**
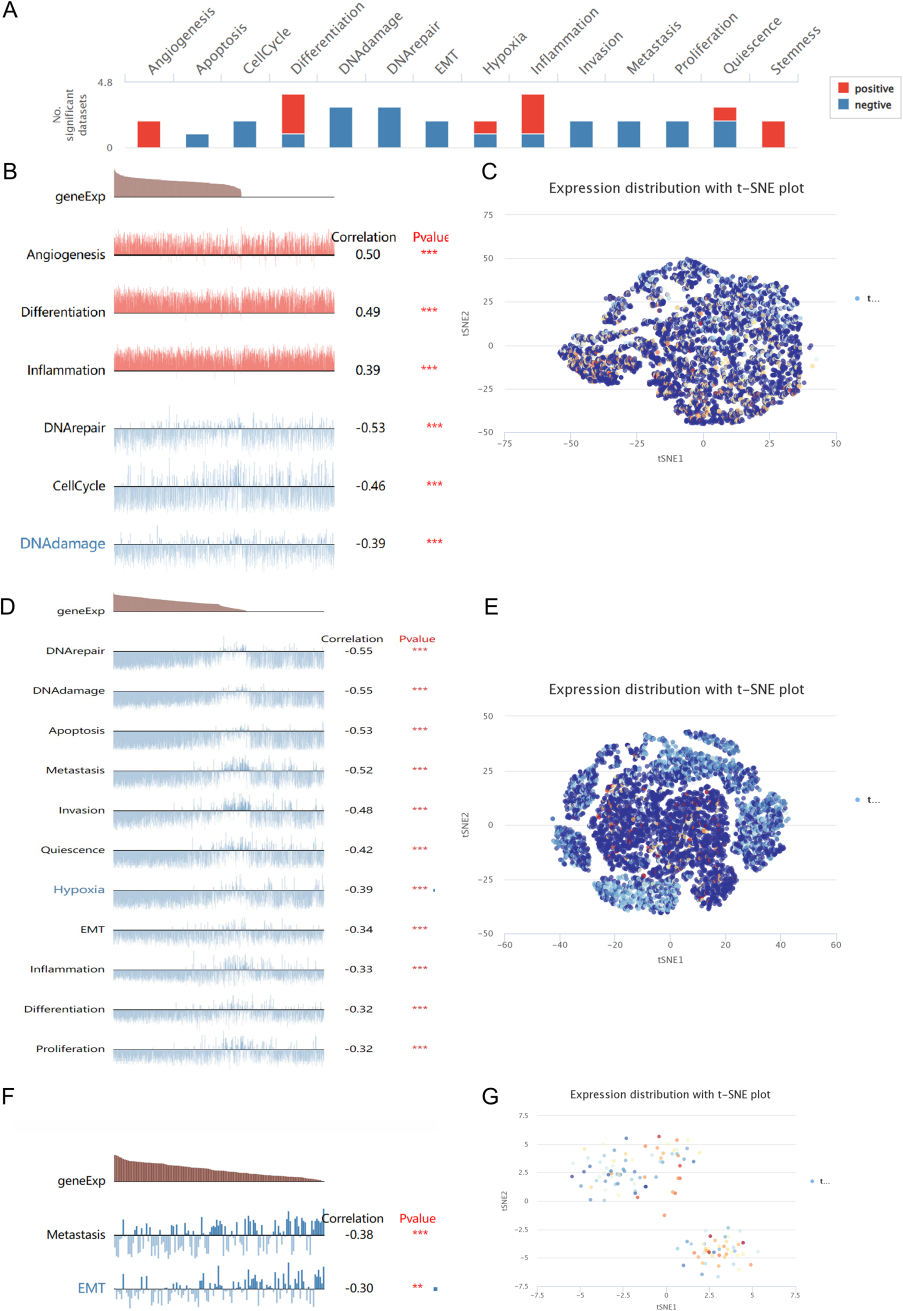
Expression level of the MPST at the single-cell level. **(A, B, D, F)** The relationship between MPST expression and different functional status in tumors was explored using the CancerSEA tool. *P < 0.05; **P < 0.01; ***P < 0.001. **(C, E, G)** The MPST expression profiles were visualized by the T-SNE plot on individual cells in RB, UM and LUAD.

Analysis of MPST correlations across 14 functional states in multiple cancers (**Figure 8A**) revealed cancer-specific patterns. In retinoblastoma (RB), MPST expression showed positive associations with angiogenesis, cell differentiation, and inflammation, but negative associations with DNA repair response, DNA damage, and cell cycle (**Figure 8B**). Uveal melanoma (UM) demonstrated negative correlations between MPST expression and examined processes, including DNA repair, DNA damage response, EMT, and angiogenesis (**Figure 8D**). Lung adenocarcinoma (LUAD) exhibited an inverse relationship between MPST expression and both metastasis and EMT (**Figure 8F**). Single-cell level visualization of MPST expression patterns in RB, UM, and LUAD was achieved through T-SNE plots (**Figures 8C, E, G**).

### 3.11 Correlation between MPST expression and DNA methylation

**Figure 9:**
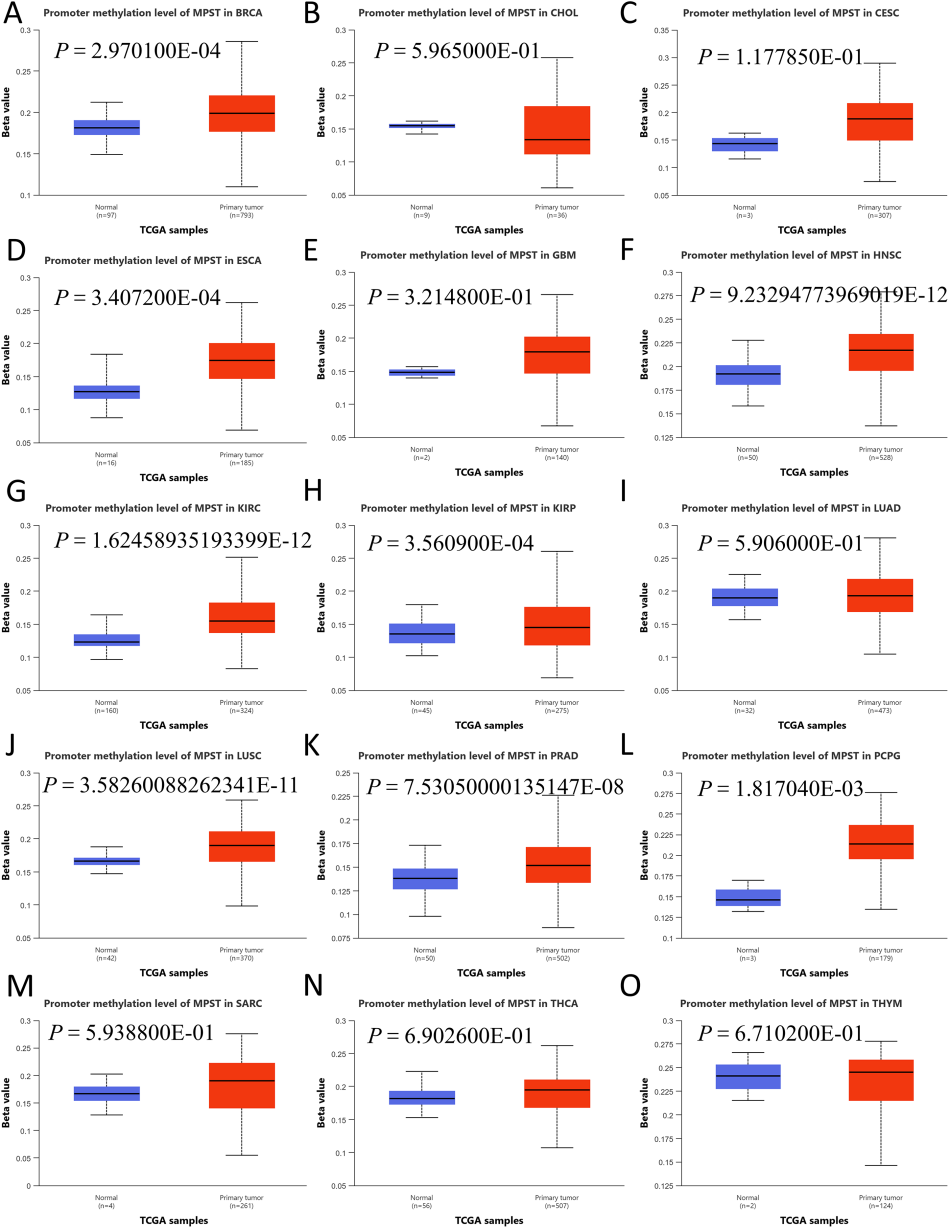
Differential methylation of the MPST promoter in 15 cancer types **(A–O)**. The panel compares MPST promoter methylation levels between tumor and normal tissue samples across the indicated cancer cohorts.

Analysis of fifteen tumor cohorts via UALCAN identified significantly heightened MPST promoter methylation relative to normal controls (**Figure 9**). The beta values, constrained between 0 (unmethylated) and 1 (fully methylated), demonstrated marked hypermethylation across these diverse cancers.

### 3.12 MPST mutations in various tumors

**Figure 10:**
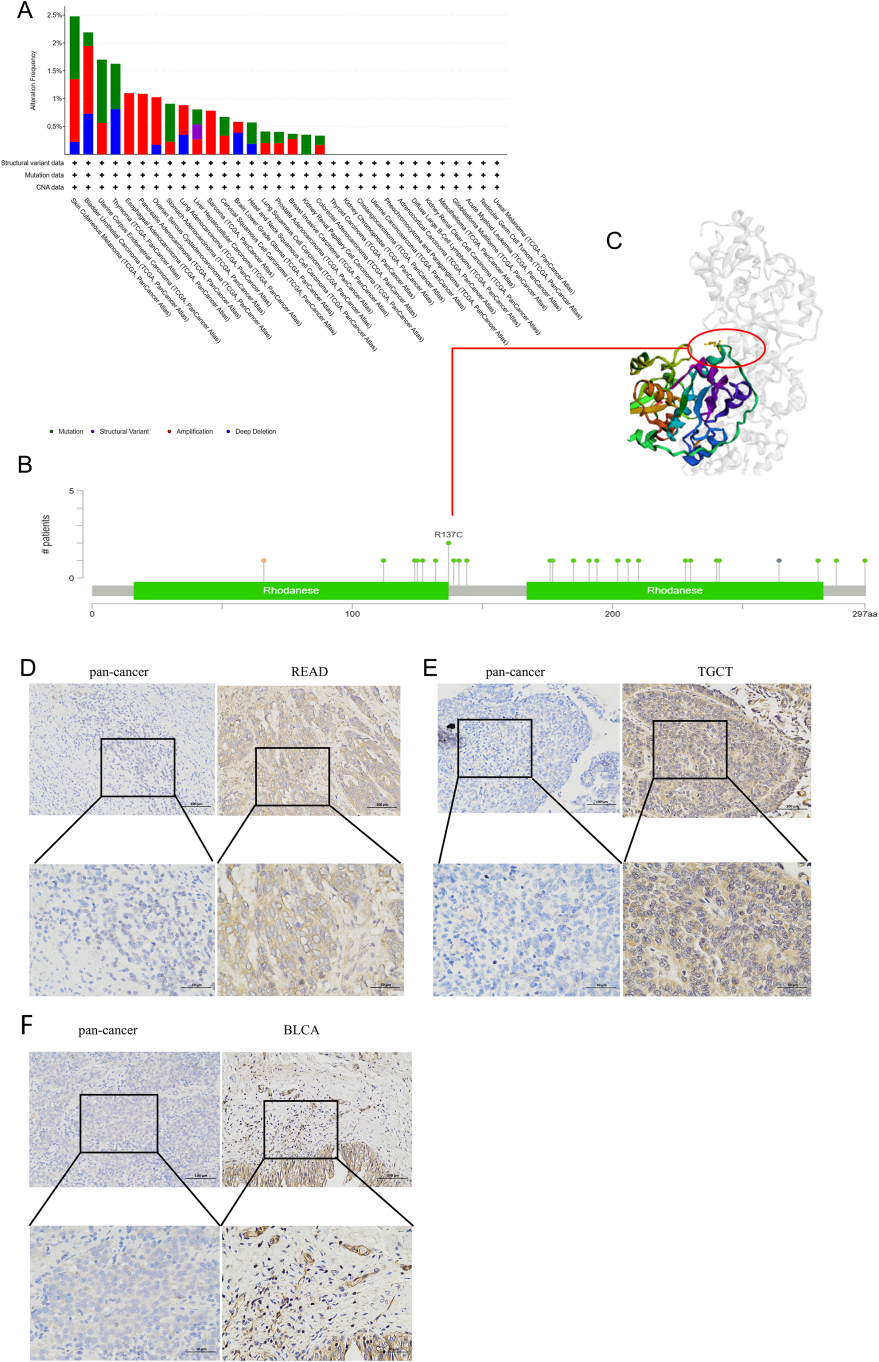
MPST gene mutations in various cancers. **(A, B)** cBioPortal shows the altered frequency of different MPST mutation types **(A)** and mutation site **(B)** in pan-cancer **(C)**. In the 3D protein structure of the MPST. The expression of MPST in the tissues was detected by immunohistochemistry: The expression of MPST in READ and pan-cancer **(D)**. The expression of MPST in TGCT and pan-cancer**(E)**. The expression of MPST in BLCA and pan-cancer **(F)**.

To characterize the genetic alterations of MPST among diverse cancer types, we analyzed its mutation profile utilizing the cBioPortal platform with data from the TCGA. Pan-cancer analysis demonstrated a higher MPST amplification rate (> 1.2%) in BLCA and a higher mutation rate in UCEC (> 1.1%). The results show that THYM exhibited the highest incidence of copy number “depth loss” at approximately 1% (**Figure 10A**). We found that missense and truncating mutations were the predominant alteration types in MPST. Among these, the R137C alteration, located in the critical Rhodanese domain, impacted a majority of the cancers studied (**Figure 10B**). The three-dimensional structure of the R137 site within MPST is illustrated in **Figure 10C**.

### 3.13 Differences in expression of MPST in READ, TGCT and BLCA

Given significant MPST-clinical correlations, BLCA, READ and TGCT were advanced to experimental validation. IHC analysis revealed intensified MPST staining density in malignant tissues relative to matched normal counterparts in READ, TGCT, and BLCA (**Figures 10D–F**).

## 4 Discussion

Cancer poses a significant and escalating threat to global health, as evidenced by China’s substantial burden of approximately 4.82 million new cases in 2022, accounting for nearly one-quarter of the global total (GLOBOCAN 2022) (29). Recently, pervasive effective biomarkers have been uncovered; however, many tumor types still lack reliable diagnostic and therapeutic targets, resulting in poor prognoses during treatment (30). Therefore, the identification and investigation of novel, more effective tumor markers could significantly improve tumor diagnosis and therapeutic strategies. In this context, mercaptopyruvate sulfurtransferase (MPST), a key enzyme for endogenous hydrogen sulfide (H_2_S) production—a gaseous signaling molecule involved in various cancer-related processes—has drawn our attention. Beyond its enzymatic function, its expression pattern suggests potential as a prognostic marker (6, 31, 32). This study is the first time to confirm that MPST gene expression levels may affect the prognosis of some cancers. In addition, the study also explores in depth the tumor immune microenvironment and its role in promoting tumor progression. Our analysis includes gene expression pathways related to cancer and single-cell molecular data, elucidating the functional roles of MPST under various conditions. Furthermore, we examine associated methylation patterns and gene mutations to clarify the genetic impact of MPST on cancer progression.

Prior research has established that MPST suppresses the advancement of hepatic carcinoma (1, 33, 34), but its expression profile across different cancer types remains unclear. This study undertakes a pioneering investigation of MPST linkage with pan-cancer datasets. We performed a systematic comparison of MPST transcriptional profiles across non-malignant versus tumor specimens using large-scale omics data derived from the TCGA and TCGA_GTEx databases across multiple organ types. Although the three datasets generally produced consistent results, discrepancies and even contradictory findings were observed, for example, in PAAD and KIRC when comparing the TCGA_GTEx and TCGA datasets. These inconsistencies likely arise from differences in control group sample sizes, highlighting the need to increase control sample sizes to achieve more robust conclusions.

MPST expression was found to correlate with clinical stages in ACC, BLCA, HNSC, and TGCT, suggesting that MPST may be correlated with tumor progression. We also noted that MPST expression within tumors was associated with tumor, age, and sex. To evaluate MPST’s prognostic relevance, we performed survival analyses across cancer types, correlating transcript levels with clinical outcomes, including OS, DSS, PFI, which was used by Kaplan-Meier curves across various cancer types. Integrating these findings, we identified that MPST had low expression in ACC, HNSC, READ, SKCM, SRAC, and TGCT and suggested poor-prognosis in patients. The most striking finding in READ was the dissociation between MPST’s association with T-stage and DFS. While lower MPST expression was linked to larger, more locally advanced tumors (T3/T4), it paradoxically predicted superior DFS. Therefore, the relationship between MPST expression levels and clinical outcomes in READ is likely multifaceted. A critical unresolved question is whether MPST expression correlates with differential responses to neoadjuvant therapy, which could potentially reconcile the observed disparity between local invasion (T-stage) and overall survival. Future studies correlating MPST with pathological response data are essential to elucidate this point. There are also studies (34) that suggested a prognostic role for MPST in certain cancers, although those studies primarily relied on bioinformatics analyses and immunohistochemistry and require further experimental validation. Taken together, these findings demonstrate that MPST has the capacity to serve as a clinically significant prognostic biomarker in a specific subset of cancers, such as ACC, BLCA, HNSC, READ, SARC, SKCM, and TGCT.

Immunity critically governs malignancy initiation and progression. Patient responses to immunotherapy are contingent upon both immune cell infiltration density and immune-related gene signatures within solid tumors (35, 36). The tumor microenvironment (TME) comprises a heterogeneous assembly of cancer cells, immune and inflammatory cells, endothelial cells, cancer-associated fibroblasts (CAF), extracellular matrix (ECM), adipocytes, neurons, and so on. Each cellular component within the TME actively influences cancer progression, rendering the TME a strategic target for therapeutic intervention (37). Accumulating evidence establishes immune cell infiltration as a critical determinant of tumor prognosis (38). We identified a significant negative correlation between MPST expression and immune infiltration metrics (ESTIMATE Score) in certain cancers (GBM, GBM, READ, TGCT, BLCA and ACC). In alignment with these findings, our study revealed immune cell infiltration levels linked to prognosis across multiple cancer types (39). Our multi-faceted analysis, integrating ESTIMATE algorithm scores with TMB, MSI, and immune checkpoint profiling, finds elevated MPST expression may represent a pivotal adaptive immune escape mechanism (21). The accumulation of a high TMB drives the presentation of neoantigens, thereby eliciting a potent T cell-mediated anti-tumor immune response (20). Collectively, our computational analyses suggest a model wherein tumors with high TMB/MSI, which typically bear high neoantigen loads and face substantial immunogenic pressure, may exhibit upregulated MPST expression. We hypothesize that this could represent an adaptive response potentially contributing to an immunosuppressive tumor microenvironment. This putative adaptation might serve as a mechanism to counteract anti-tumor immunity, which could influence tumor survival. The observed negative correlation between MPST and immune checkpoint molecules like CTLA-4 in certain cancers is consistent with this notion (40). It raises the possibility that MPST upregulation could be part of an immune modulatory strategy, perhaps attenuating T-cell activity upstream and thereby altering the context for checkpoint inhibition. These associations position MPST as a candidate molecule of interest in tumor immunology, meriting further investigation to assess its potential as a target for novel immunotherapy strategies. However, these predictions require validation with spatial proteomic techniques to determine if MPST-high regions in tumors are indeed characterized by specific immune cell exclusion or attraction. If experimentally confirmed, understanding MPST’s role in shaping the immune landscape could inform the development of novel immunotherapeutic strategies.

Traditional biotechnological analysis can only analyze the differences between the study samples from a comprehensive viewpoint, and the changes shown are insufficient to characterize the variations between individual cells (41). Consequently, we leveraged the CancerSEA database to interrogate single-cell resolution associations between MPST expression and functional states across cancers.

In RB, MPST expression negatively correlates with DNA damage, cell cycle progression, and DNA repair responses, but shows positive links to angiogenesis, cellular differentiation, and inflammatory activity. In UM, almost all tumor biological characteristics, including angiogenesis, DNA damage response, DNA repair, and epithelial-mesenchymal transformation (EMT), are negatively correlated with MPST. This demonstrates that in certain malignancies, we can modify MPST expression to alter the expression of functions connected to tumor cells *in vivo*, hence influencing the tumor prognosis and possibly using MPST as a potential target for therapeutic intervention.

To define the functional role of MPST in cancer, we selected its top 100 co-expressed genes for protein-protein interaction (PPI) network construction and gene set enrichment analysis (GSEA). GO and KEGG analyses of the PPI network revealed its association with cancer-related pathways, including the PPAR signaling pathway, enzyme inhibitor activity, and glycine, serine, and threonine metabolism. Furthermore, GSEA indicated that MPST-linked genes are enriched in various tumors and immune-related processes, such as eukaryotic translation elongation, FCGR activation, and calcium mobilization, underscoring its broad influence on tumor development.

Subsequently we explored the MPST in terms of methylation and gene mutation (37, 42). Genetic alterations in MPST are found in the majority of cancers, with a wide range of mutation sites. The highest mutation rates are observed in BLCA, UCEC, and THYM. Our study also reveals that the types and distribution of gene mutations in MPST vary across different cancers. Additionally, missense mutations and truncations are the primary contributors to cancer development in MPST. Therefore, addressing these two types of genetic changes could potentially aid in cancer diagnosis. DNA methylation of cytosines is a pivotal alteration in controlling gene activity. Dysregulated methylation profiles are often associated with the derangement of regulatory mechanisms in cancerous cells (43–46). This study has uncovered a paradoxical relationship between elevated methylation levels of the MPST promoter and increased MPST expression in tumor tissues. This study explores multiple mechanisms that may account for this phenomenon, such as the binding of transcription inhibitors that inadvertently promote gene activation (47), the involvement of remote control elements, and the effects of alternative promoter activation (48), are discussed in detail by Smith et al. This study shows that the complexity of gene regulation networks, revealing that methylation can sometimes enhance gene activity in a tumorigenic context.

Nevertheless, several limitations of this study warrant consideration. Primarily, our analysis relies on existing online databases rather than primary data collection. To mitigate this limitation, we implemented a rigorous cross-validation methodology utilizing multiple databases to enhance the reliability of our findings. Furthermore, our analysis of immune cell infiltration is based on in silico deconvolution algorithms, and these predictions require validation with spatial proteomic techniques. Additionally, this study correlates MPST expression with clinical outcomes but does not include experimental validation of its enzymatic output. Regarding functional enrichment, we have identified potential regulatory pathways involving MPST, but have not elucidated the specific molecular mechanisms within these pathways. Given the paucity of published research in this domain, our study serves primarily to establish foundational knowledge and suggest novel research directions. This study has achieved clinical validation through immunohistochemistry and plans to further verify the computational findings using *in vitro* and *in vivo* experiments in future research. One key limitation is that the precise functional mechanisms of MPST, particularly its enzymatic output, have not been explored via *in vitro* assays, primarily due to constraints in time and research resources. Therefore, future work should directly measure cellular H_2_S levels in models with modulated MPST expression to confirm the functional link between its expression, H_2_S production, and the observed aggressive phenotypes.

## 5 Conclusion

Pan-cancer analysis revealed that MPST expression is significantly dysregulated in tumor tissues compared to normal controls, a finding that has been preliminarily validated. Furthermore, MPST expression associated with clinical prognosis and the tumor immune microenvironment, as evidenced by its association with multiple immune cell types. From a single-cell perspective, MPST likely influences tumorigenesis through its involvement in DNA repair, and immune-related pathways, including Fcgr activation. Moreover, DNA methylation and gene missense mutations in MPST may independently promote tumor development. Therefore, MPST is a potential therapeutic target, a useful diagnostic and diagnostic indicator, and also an opportunity to develop novel immunotherapeutic advancements.

## Data Availability

The original contributions presented in the study are included in the article/**Supplementary Material**. Further inquiries can be directed to the corresponding authors.

## Supplementary material

Supplementary Figure 1 The relation between MPST expression and DSS in pan-cancer. **(A)** Effect of MPST expression on the pan-cancer DSS. **(B–F)** Effect of MPST expression on DSS in ACC, BLCA, HNSC, READ, and SKCM, respectively.

Supplementary Figure 2 The relation between MPST expression and PFI in pan-cancer. **(A)** Effect of MPST expression on the pan-cancer PFI. **(B–G)** Effect of MPST expression on PFI in ACC, BLCA, GBM, SKCM and TGCT, respectively.

Supplementary Figure 3 The AUC of the ROC curve validated the diagnostic performance of the MPST in the TCGA cohort **(A–F)**.

Supplementary Figure 4 Nomogram models were established in ACC, SARC. **(A)** A nomogram model containing MPST expression in ACC. **(B)**The nomogram model was used to evaluate the ACC at 1 year, 3 year and 5 years. **(C)** A nomogram model containing MPST expression in SARC. **(D)**The nomogram model was used to evaluate the SARC at 1 year, 3 year and 5 years.

Supplementary Figure 5 Analysis of MPST expression in the tumor immune microenvironment was evaluated using the ESTIMATE algorithm. **(A–H)** Correlations between MPST expression and the Stromal Score in eight cancer types (GBM, SARC, HNSC, READ, SKCM, TGCT, BLCA, ACC). **(I)** Correlation of MPST expression with tumor mutational burden (TMB) across various cancer types. **(J)** Correlation of MPST expression with microsatellite instability (MSI) across various cancer types. Cancer types significantly associated with patient prognosis are highlighted with red borders.
