# Supplementary figures and images for: The expression of mercaptopyruvate sulfurtransferase serves as a potential biomarker for prognostic stratification and immunotherapy in certain cancers
Source: Front Oncol. 2026 Jan 14;15:1686443. doi: 10.3389/fonc.2025.1686443 (PMC12847049; doi:10.3389/fonc.2025.1686443)

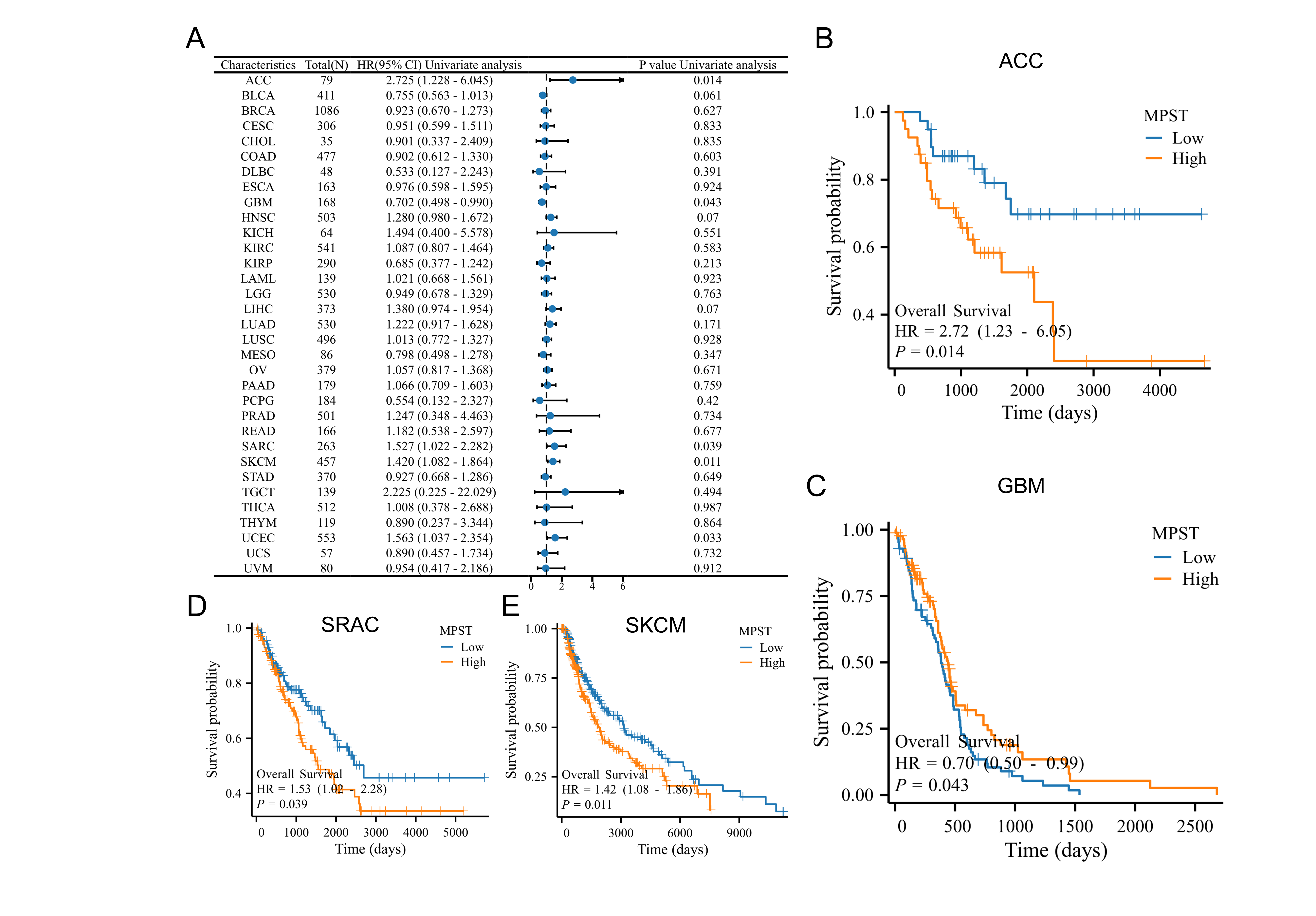

Supplement: Supplementary Figure 1 — The relation between MPST expression and DSS in pan-cancer. (A) Effect of MPST expression on the pan-cancer DSS. (B–F) Effect of MPST expression on DSS in ACC, BLCA, HNSC, READ, and SKCM, respectively. [file Image1.tif]

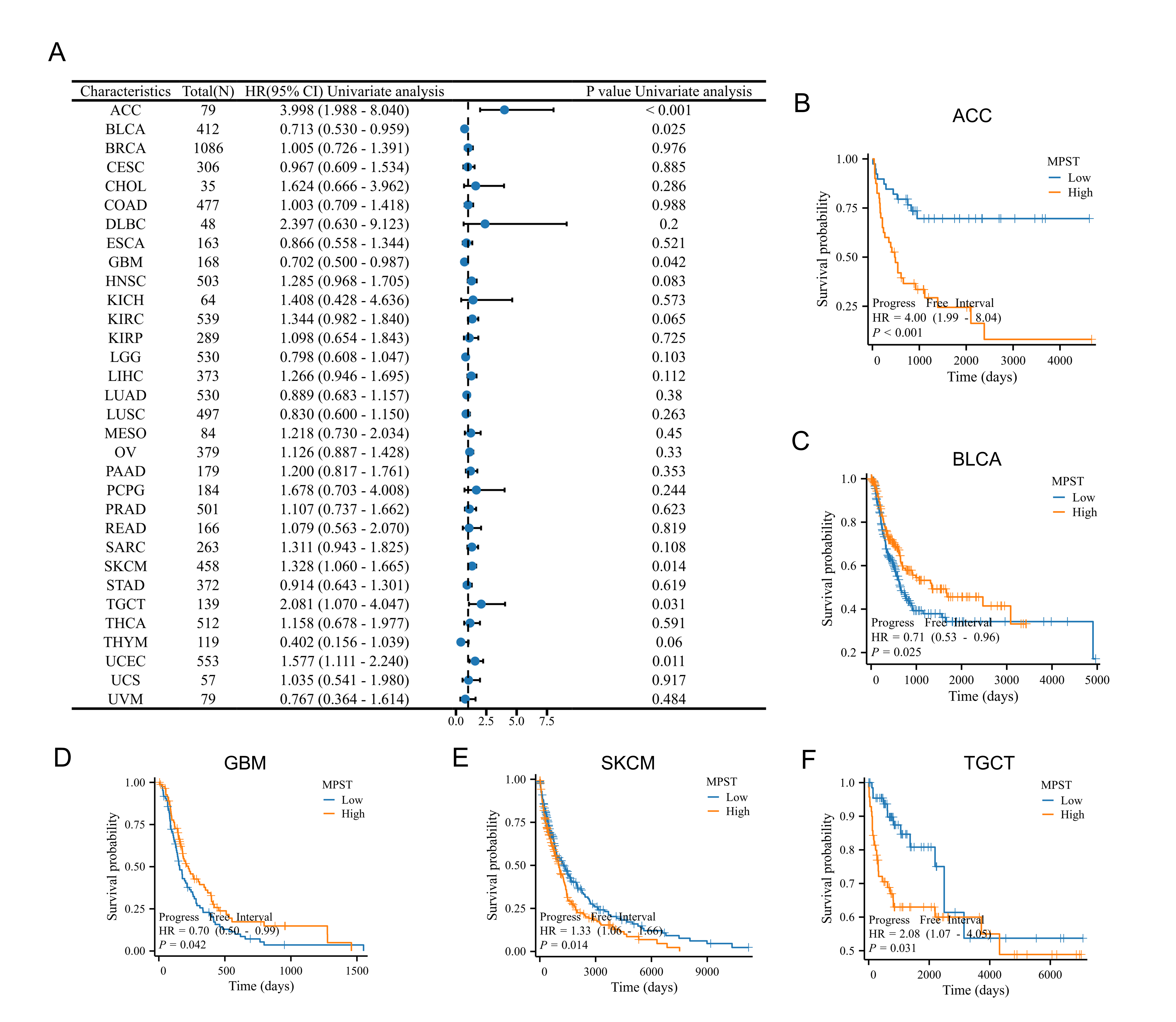

Supplement: Supplementary Figure 2 — The relation between MPST expression and PFI in pan-cancer. (A) Effect of MPST expression on the pan-cancer PFI. (B–G) Effect of MPST expression on PFI in ACC, BLCA, GBM, SKCM and TGCT, respectively. [file Image2.tif]

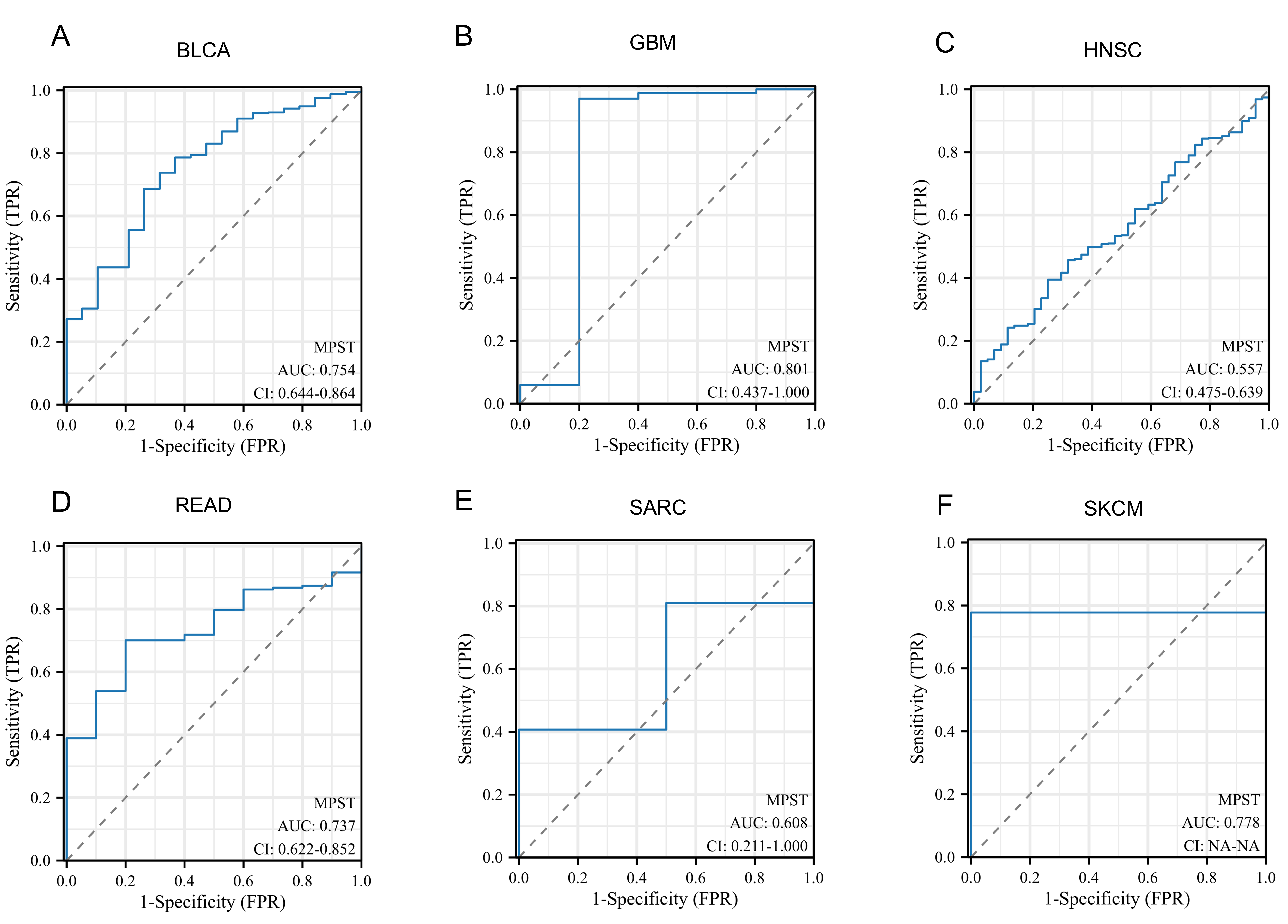

Supplement: Supplementary Figure 3 — The AUC of the ROC curve validated the diagnostic performance of the MPST in the TCGA cohort (A–F). [file Image3.tif]

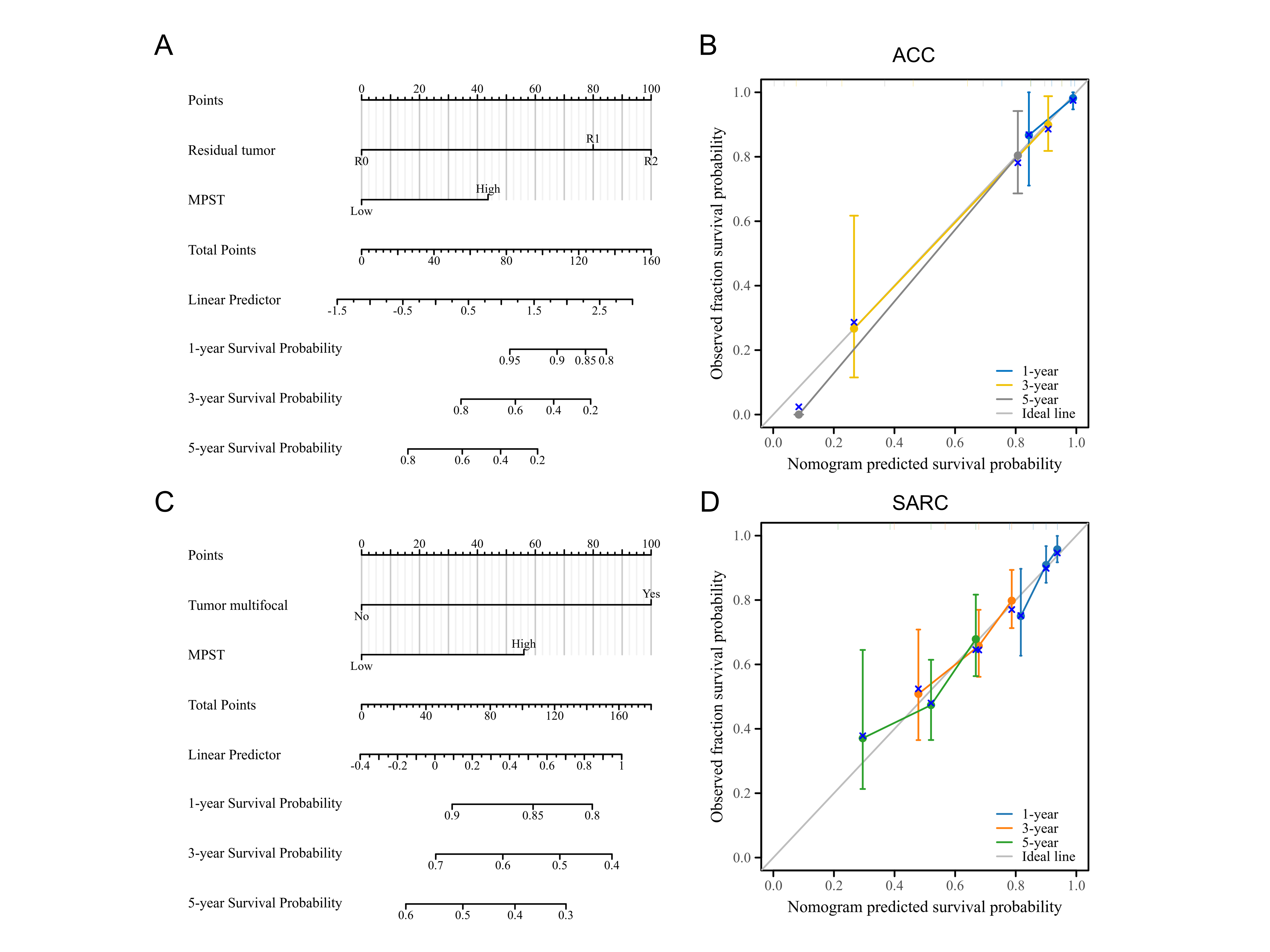

Supplement: Supplementary Figure 4 — Nomogram models were established in ACC, SARC. (A) A nomogram model containing MPST expression in ACC. (B)The nomogram model was used to evaluate the ACC at 1 year, 3 year and 5 years. (C) A nomogram model containing MPST expression in SARC. (D)The nomogram model was used to evaluate the SARC at 1 year, 3 year and 5 years. [file Image4.tif]

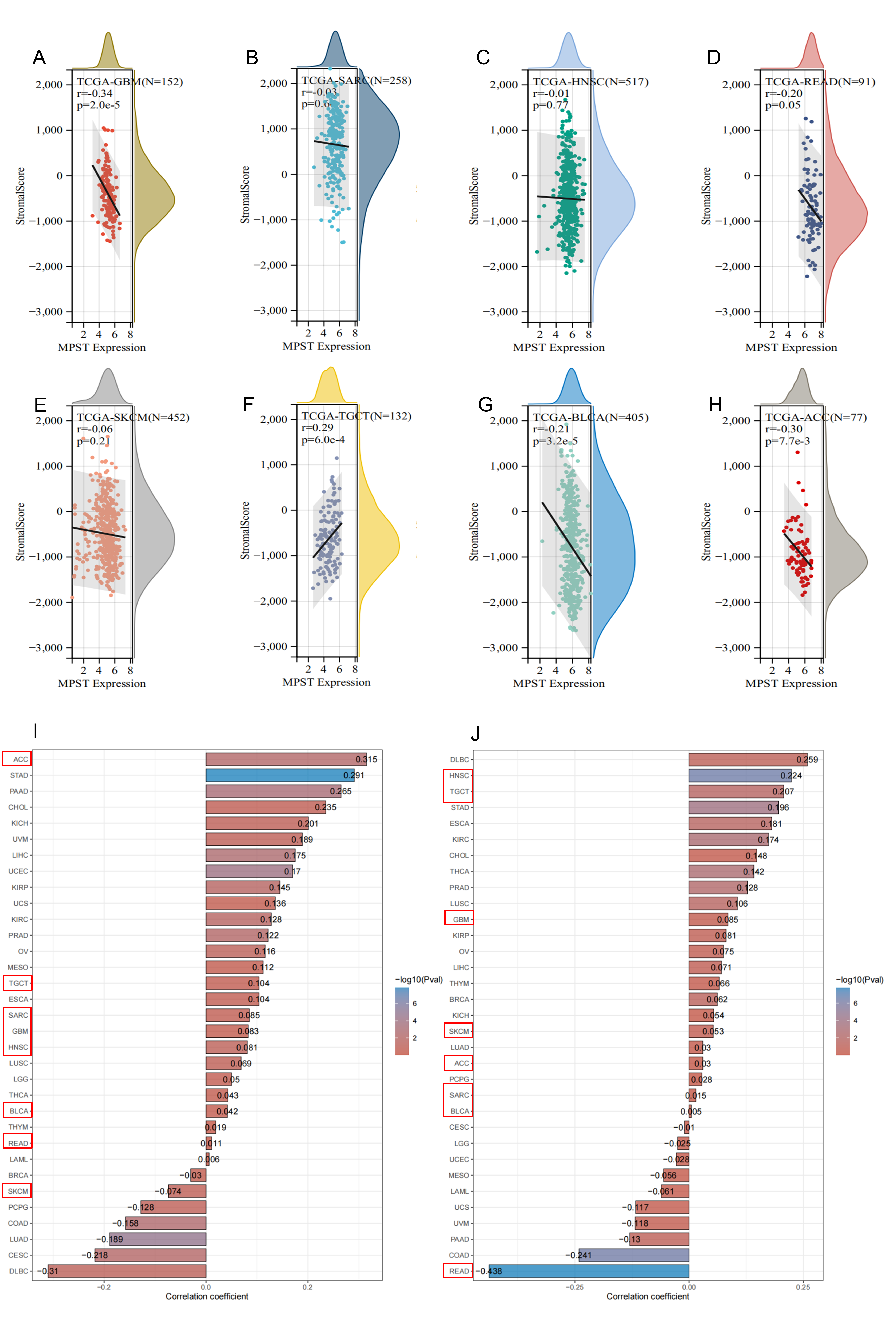

Supplement: Supplementary Figure 5 — Analysis of MPST expression in the tumor immune microenvironment was evaluated using the ESTIMATE algorithm. (A–H) Correlations between MPST expression and the Stromal Score in eight cancer types (GBM, SARC, HNSC, READ, SKCM, TGCT, BLCA, ACC). (I) Correlation of MPST expression with tumor mutational burden (TMB) across various cancer types. (J) Correlation of MPST expression with microsatellite instability (MSI) across various cancer types. Cancer types significantly associated with patient prognosis are highlighted with red borders. [file Image5.tif]
